# Public Oral Health Care During COVID-19: Time for Reflection and Action

**DOI:** 10.3389/fmed.2021.610450

**Published:** 2021-03-17

**Authors:** Shenuka Singh

**Affiliations:** Discipline of Dentistry, School of Health Sciences, University of KwaZulu-Natal (UKZN), Durban, South Africa

**Keywords:** public sector, oral health promotion, COVID-19, planning, integrated health

## Introduction

About 2.3 billion people (mainly from upper and lower middle income countries) suffer from untreated dental caries (affecting permanent teeth) while 530 million children have caries involving primary teeth, according to the Global Burden of Disease 2017. This makes dental caries one of the most common global public health concerns (1, 2). Data from the Global Burden of Disease 2017 further suggest a skewed distribution of reported oral diseases across the global platform. Countries with better economic development have a higher burden of tooth loss yet lower rates of untreated caries and severe periodontitis (3). Changes in dietary intake, increased sugar consumption, and limited access to health care could further be attributed to the reported rates of diabetes, obesity, and dental caries (3). Similarly, increasing rates in periodontal disease, oral cancers, oral manifestations of HIV, and oro-dental trauma demand a more committed public health response (1). Oral diseases are considered a major burden on scarce resources, especially from a public health perspective, due to their impact on pain, discomfort, and compromised quality of life (4, 5). Treatment for oral conditions are generally unaffordable and universal health coverage remains a challenge in most parts of the world. Even in high income countries, oral health related care amounts to almost 5% of the total health expenditure and 20% out-of-pocket health expenditure (1).

The COVID-19 pandemic has sharply brought into focus the need to re-examine oral health systems given that virtually every aspect of healthcare delivery has been impacted by the outbreak including oral health care delivery. It can be argued that COVID-19 has completely altered the care landscape with not only new challenges and threats, but also new opportunities. Likewise dentistry and oral care delivery should not be left behind as the entire health delivery systems morphs and evolves along with the times. This paper advocates a review of public oral health systems (both in developed and developing countries) with a renewed focus on improving oral health related outcomes, as a response to the COVID-19 pandemic. A SWOT analysis framework is used to examine strengths, weaknesses, opportunities, and threats related to the current delivery of oral health services. The strengths and weaknesses of oral health systems will first be examined, followed by opportunities to improve oral health care delivery. Possible threats to achieving improved service delivery will also be explored. This analysis is intended to create greater awareness among oral health and health practitioners, oral health planners, policy makers, health decision-makers, researchers, and academics on the value of oral health within general health care. Additionally, this analysis could be used to guide oral health related “decision-making as it is crucial during the current pandemic to work on weaknesses, avoid threats, and utilize all future opportunities” [(6), p.1343].

## Strengths and Weaknesses of Oral Health Systems

Oral health systems (both public and private) have been in existence but the challenges of delivering effective oral health care remains a challenge in many parts of the world (4), especially in underserved populations. Given the dependency on the public oral health system, especially in economically challenged countries and in under-served populations (7), it is imperative that the health system is able to respond adequately to unmet oral health needs. While the link between oral health and general health has been widely documented, the challenges of implementing an integrated response to health care also continue to persist in several health settings (8). One of the main shortcomings of oral health care is that it is generally assessed independently of general health outcomes while measures of overall health status tend to exclude oral health determinants (9). Another shortcoming would be a dependency on the formal health system to deliver optimal oral health care, without taking into account other key platforms such as community or facility-based care. This divide between oral health and general health continues to permeate through health policy planning and implementation.

Other weaknesses of the health system became more apparent during COVID-19. The pre-COVID inequities in access to optimal health care have been exacerbated during the pandemic where underserved populations and uninsured as those in the US, have been disproportionately affected by the disease (10). Similarly in Africa and other in developing countries, the rhetoric between saving lives and livelihoods becomes more pronounced as people struggle between disease prevention and earning an income (11). Concurrently, oral health systems appeared to use a “knee jerk” approach in dealing with the pandemic. While oral health authorities in some countries called for a suspension of clinical service delivery while others promoted a more cautious approach with emphasis on increased infection control (12). This sadly suggests that current country specific policies and guidelines on oral health care have failed to plan adequately for a public health emergency.

## The Need for Committed Focus On Oral Health Care

A review and re-organization of oral health systems is thus overdue. There is need to ensure equitable and improved access to oral health care with a focus on improving population health outcomes (13). There is need for increased policy discussions on oral health care, research and innovation, monitoring, and surveillance. The primary health care model could provide a viable vehicle (8) to facilitate political commitment from planning to implementation through local legislative efforts.

While there is currently no natural immunity against SARS-CoV-2 infection, studies show that people more at risk of developing COVID-19 related complications include those with diabetes and obesity (14, 15). While vaccines are now available, vaccine nationalism and allegations of stockpiling among high income countries have fuelled global discussions on addressing COVID-19 challenges and low income countries are largely left with little bargaining power (16). The emergence of new variants in COVID-19 has made the disease far more transmissible, thereby placing a greater strain on the health system (17). At the same time, diet and obesity are linked to acquisitions of unhealthy lifestyle practices that are deeply rooted in social and economic circumstances (18–20), that could limit the selection of healthier choices. These determinants could thus impact on oral health status as well (21). Concurrently, access to oral care for people afflicted with COVID-19 disease could be compromised due to ongoing restrictions in social movement during state imposed lockdowns.

Hence a more committed focus is needed on prevention of commonly occurring oral diseases and the promotion of healthier behavioral practices. Isolated individual interventions directed toward modifying specific oral health-related behaviors have not been successful in achieving long-term changes in behavioral practices (5). A population based strategy could include a multi-disciplinary approach for the reduction of risk factors associated with tobacco and alcohol use, healthier dietary intake, and exposure to additional topical fluoride within wider integrated health action. The availability and cost of healthier foods as well as providing information on food labels could influence food choices (22).

## Opportunities for Improved Oral Health Care Deliver

Despite the highlighted challenges, several opportunities exist to optimize a more seamless delivery of oral health services, as outlined below:

### Oral Health Promotion

Promotion of oral health care should not be the responsibility of just the health system. From an organizational perspective this requires a shift from health practitioner focus to oral health promotion within a social setting (21, 23). It is known that oral health self-care emanates from the primary socialization period, that is, children engage in activities such general hygiene and taking care of one's oral cavity starts through influences within the family and social setting (22). Oral health self-care can be reinforced at multiple settings such as crèches, schools, workplaces, etc. Oral health action could include efforts to modify oral health behaviors through oral health education, dietary counseling, individual skills development (such as proper toothbrushing and flossing) and access to additional fluoride uptake. In addition, awareness programmes to control risks to oral health such as nutrition intake, tobacco cessation, reduction in alcohol consumption, and avoidance of dental trauma should be considered. Other initiatives could include access to clean water and a safe and supportive environment to develop personal skills for positive oral health outcomes (4, 5, 8, 21, 22). These activities should be integrated into COVID-19 public health measures such as regular handwashing, social distancing, and wearing of face masks. Policy development at the facility or health promotion setting level could assist in ensuing commitments in implementing these initiatives. Oral health promotion delivery needs to occur at multiple levels such as macro level (policy development and agenda setting), meso level (community-based approach), and micro level (interpersonal and intrapersonal). A macro level engagement could also include mass media communications and foster political support for creating supportive environments to address unhealthy behavioral practices (21). Mass media provides opportunities to advocate for integrated health and social messages during COVID-19 that include elements of oral health care (24). However, caution should also be applied to prevent spread of fake news and health-related mis-information (25).

Further investments in oral health could include engagement with technologies to improve the digital interface in health communication (26). Modalities such as tele-dentistry could support the delivery of oral health services, specifically when physical contact or travel to the local clinic is difficult. Tele-dentistry also has potential to link service providers across geographical spaces and ensure continuity in the delivery of expertise in oral health care (26–28). Health or oral health care workers could use these technologies to facilitate support and engagement with service providers in care homes, correctional service facilities and other such settings where public access during COVID-19 is limited. This could ensure that integrated oral health messages and support can still reach these very vulnerable populations. Similarly, remote engagement provides another viable platform for the dissemination of preventive messages. Mobile devices such as smartphones create a personalized platform for the delivery of oral health messages. Although issues such as internet connectivity and availability of data bundles remain a challenge in resource-constrained settings, it is nevertheless valuable to invest in such technologies.

Likewise, stronger collaboration is required between the health system and community based settings for the safe delivery of oral health promotion services (29). These settings provide further opportunities for integrated oral health messages and policy planning (30) such as good oral health habits, positive oral health behaviors, and developing personal oral health skills. Oral health care should be embraced by all stakeholders involved in care provision, and not just the health or oral health worker. Partnerships in health care could also be developed with the private sector and all other stakeholders in community development (29, 30). Thus, the prevention of disease and promotion of healthier lifestyles are integrated into overall community and social development.

Oral health interventions directed at the intrapersonal and interpersonal level should focus on unhealthy behavioral practices such as diet, smoking and alcohol consumption. Smoking has been shown to be linked to a number of health complications such as diminished lung capacity, cardiac complications, and periodontal disease (31). Further complications can linked with COVID-19 (32) given that this a respiratory related disease. Integrated oral health messages could thus be directly beneficial at an individual level. Public health emergency strategies such as isolation and quarantine during COVID-19 places additional strain on the individual's ability to cope with the pandemic (33). Strategies could be developed at the micro-level to help individuals cope with COVID-19, and make healthier behavioral choices. Oral health care workers have a role to play in early recognition of signs of depression in patients and to make the necessary referrals for appropriate care.

Studies have shown that early clinical signs of COVID-19 include a loss of taste and smell (34, 35). COVID-19 provides unique opportunities for oral health care workers to engage in screening services. Thus, oral health workers could play key roles in screening (36) and identifying asymptomatic clients or those presenting with early symptoms; and conduct saliva testing for COVID-19 (37). Oral health care workers could provide integrated health messages such as handwashing, cough etiquette, proper wearing of facial masks/coverings, and oral health related care. Other activities could include infection prevention education and training, reinforcing health and safety measures in client/patient contact, risk management, and program evaluation (12).

### Threats

Despite these opportunities some of the possible threats to implementing these strategies could include lack of practitioner skills. There is thus a need for continuing professional and skills development for the oral health care worker as well as a review of undergraduate dental curricula so that appropriate training can provided to better equip the individual practitioner.

Although the notion of integrated oral health care through a multi-disciplinary team approach has been touted as a key strategy to broaden the delivery of oral health promotion services (38), this seemingly uncomplicated patient/client centered initiative continues to face challenges in many parts of the world, as indicated earlier (39, 40). The question begs: why is this so? Perhaps we as dental public health practitioners, academics, health planners, and researchers need to reflect further. Has there been widespread stakeholder engagement to ensure buy-in for these innovative approaches in oral health service delivery? To successfully influence the processes of oral health promotion requires more than simple, document-based policy reforms that are strong on rhetoric, and good ideas, but have not achieved the widespread stakeholder support necessary to carry them through to funding and implementation [(41), p.23]. Simultaneously, we as oral health care workers need to re-examine our roles within a multi-disciplinary team. How can we make more meaningful contribution to overall health outcomes during COVID-19 (42)?

This highlights the need for greater dialogue both within and outside of the health system to explore ways of integrating oral health messages and care within a broader scope of overall community health. This should be supported by ongoing opportunities for health workers to develop skills in oral health promotion activities through training programmes. Further research is required to unpack the barriers and delays in integrated health action.

## Conclusion

The COVID-19 pandemic has provided opportunities for renewed interest in the oral health agenda. A re-organization of oral health systems with a focus on social orientation of oral health self-care, could see improved oral health gains in the long term. Concurrently, the role of oral health care workers and their preparedness for a public health emergency should be reviewed and addressed through appropriate training platforms.

## Author Contributions

The author confirms being the sole contributor of this work and has approved it for publication.

## Conflict of Interest

The author declares that the research was conducted in the absence of any commercial or financial relationships that could be construed as a potential conflict of interest.
